# Wound healing in db/db mice with type 2 diabetes using non-contact exposure with an argon non-thermal atmospheric pressure plasma jet device

**DOI:** 10.1371/journal.pone.0275602

**Published:** 2022-10-14

**Authors:** Arya Iswara, Kenta Tanaka, Tatsuo Ishijima, Yukari Nakajima, Kanae Mukai, Yasunori Tanaka, Yusuke Nakano, Junko Sugama, Makoto Oe, Mayumi Okuwa, Toshio Nakatani

**Affiliations:** 1 Division of Health Sciences, Department of Clinical Nursing, Graduate Course of Nursing Science, Graduate School of Medical Sciences, Kanazawa University, Ishikawa, Japan; 2 Department of Medical Laboratory Science, Faculty of Nursing and Health Sciences, Universitas Muhammadiyah Semarang, Semarang, Indonesia; 3 Division of Electrical, Information and Communication Engineering Graduate School of Natural Science and Technology, Kanazawa University, Kanazawa, Ishikawa, Japan; 4 Faculty of Electrical, Information, and Communication Engineering, Institute of Science and Engineering, Kanazawa University, Kanazawa, Ishikawa, Japan; 5 Faculty of Health Sciences, Institute of Medical, Pharmaceutical and Health Sciences, Kanazawa University, Ishikawa, Japan; 6 Research Center for Implementation Nursing Science Initiative, School of Health Sciences, Fujita Health University, Aichi, Japan; Universite de Technologie de Compiegne, FRANCE

## Abstract

A non-thermal atmospheric pressure plasma jet (APPJ) may stimulate cells and tissues or result in cell death depending on the intensity of plasma at the target; therefore, we herein investigated the effects of non-thermal plasma under non-contact conditions on the healing of full-thickness wounds in diabetic mice (DM+ group) and normal mice (DM- group). A hydrogen peroxide colorimetric method and high performance liquid chromatography showed that APPJ produced low amounts of reactive oxygen and nitrogen species. Ten-week-old male C57BL/6j mice with normal blood glucose levels (DM- group) and 10-week-old male C57BLKS/J Iar-+Leprdb/+Leprdb mice (DM+ group) received two full-thickness cutaneous wounds (4 mm in diameter) on both sides of the dorsum. Wounds were treated with or without the plasma jet or argon gas for 1 minute and were then covered with a hydrocolloid dressing (Hydrocolloid), according to which mice were divided into the following groups: DM+Plasma, DM+Argon, DM+Hydrocolloid, DM-Plasma, DM-Argon, and DM-Hydrocolloid. Exudate weights, wound areas, and wound area ratios were recorded every day. Hematoxylin and eosin staining was performed to assess re-epithelialization and α-SMA immunohistological staining to evaluate the formation of new blood vessels. Non-thermal plasma under non-contact conditions reduced the production of exudate. Exudate weights were smaller in the DM+Plasma group than in the DM+Hydrocolloid and DM+Argon groups. The wound area ratio was smaller for plasma-treated wounds, and was also smaller in the DM+Plasma group than in the DM+Hydrocolloid and DM+Argon groups on days 1–21 (p<0.01). Wound areas were smaller in the DM-Plasma group than in the DM-Argon group until day 14 and differences were significant on days 1–5 (p<0.01). The percentage of re-epithelialization was significantly higher in the DM+Plasma group than in the DM+Argon and DM+Hydrocolloid groups (p<0.01). The number of new blood vessels that had formed by day 7 was significantly higher in the DM+Plasma group than in the DM+Hydrocolloid and DM+Argon groups (p<0.05). These results indicate that treatment with the current non-thermal plasma APPJ device under non-contact conditions accelerated wound healing in diabetic mice.

## Introduction

Wound healing as a response to injury is a natural process involving a cascade of complex and interactive cellular and biochemical processes including inflammation, proliferation, and the migration of various cell types, which ultimately results in tissue regeneration and remodeling. Tissue repair is accelerated or inhibited by matrix synthesis, collagen deposition leading to re-epithelialization, neovascularization, the formation of granulation tissue, and growth factors and cytokines [1]. The most common complication of diabetes mellitus is delayed wound healing, which is attributed to hyperglycemic conditions and leads to chronic complications [2]. Insulin resistance, impaired glucose tolerance, and severe inflammation caused by neutrophil infiltration may also contribute to delayed wound healing [3]. Previous studies demonstrated that high glucose levels had a negative impact on growth factors, such as insulin-like growth factors and vascular endothelial growth factors (VEGF), resulting in poor re-epithelialization or angiogenesis [4, 5].

In the past decade, the use of a non-thermal atmospheric pressure plasma jet (APPJ) in medical applications, particularly wound healing, has been attracting increasing attention [6, 7]. Non-thermal atmospheric pressure plasma is a source of reactive oxygen and nitrogen species (RONS) [8]. Hydrogen peroxide (H_2_O_2_), a well-known reactive oxygen species (ROS), is a second messenger of tissue growth factor, platelet-derived growth factor (PDGF), and VEGF production in wound healing. H_2_O_2_ also plays important roles in blood coagulation, wound contraction, and bacteria inactivation. Nitric oxide (NO) is another critical species in wound healing for angiogenesis, inflammation, and tissue remodeling [9].

Although RONS are key players in wound healing, a recent study demonstrated that the medical and biological effects of RONS were dependent on the environmental conditions of living cells. The impact of plasma exposure may stimulate cells and tissues or result in cell death depending on the intensity of plasma at the target, which is consistent with the oxidative stress theory. Sen et al. [10] reported that numerous aspects of wound healing were subject to biological redox control. ROS, such as H_2_O_2_, and reactive nitrogen species (RNS), including NO, are key factors influencing these mechanisms. Therefore, oxidative stress includes oxidative eustress and oxidative distress depending on low or high oxidant exposure. Oxidative eustress triggers pathways that promote proliferation, angiogenesis, migration, and re-epithelialization, which collectively enhance wound healing. In contrast, oxidative distress activates pathways that induce senescence, apoptosis, or necrosis as well as immune responses and, thus, may be beneficial for cancer treatment. Previous studies suggested that plasma treatments stimulated either tissue regeneration or cell death [11, 12]. In the present study, we used normal and diabetic mice to evaluate the wound healing effects of plasma treatment because, in contrast to normal mice, the healing process is impaired in diabetic wounds. The effects of plasma treatment on wound healing were also examined in normal mice. We used an APPJ device developed by our research group. The previous APPJ device was limited by its static placement, its high consumption of the feeding gas, and direct contact of the plasma plume with the wound area. We developed a handheld APPJ device, which allows it to be easily maneuvered around the wound area. It also uses a lower feeding gas flow and the plasma bullet generated inside the tube prevents direct contact of the plasma plume with the wound area. Our developed APPJ device, which treats wounds under non-contact conditions and produces less RONS, may promote the healing of full-thickness wounds under normal or diabetic conditions.

## Materials and methods

### APPJ device

**Fig 1:**
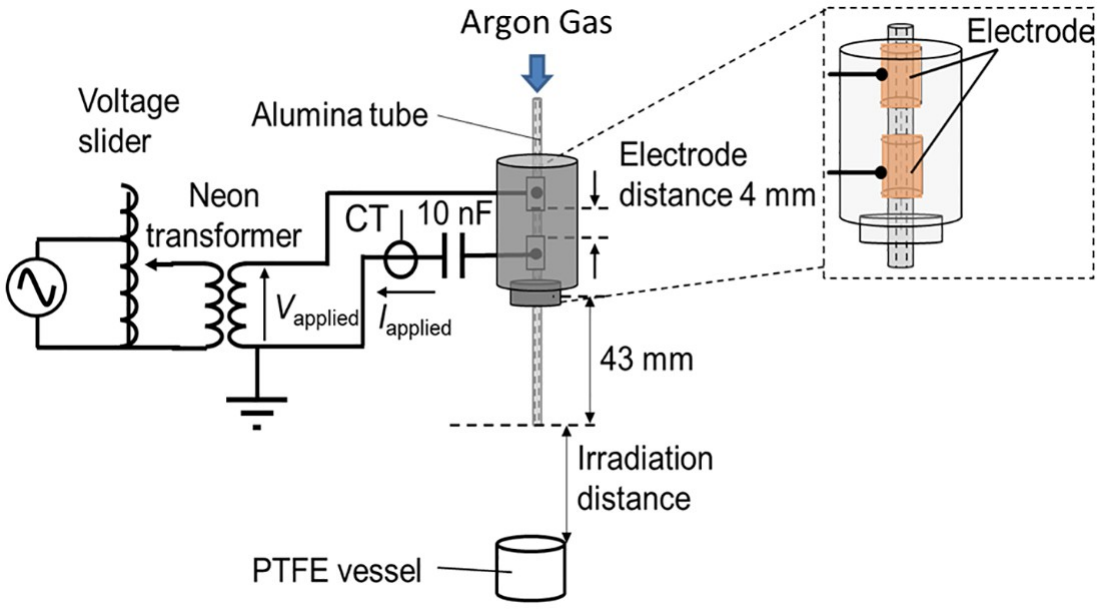
Experimental set-up of the atmospheric pressure plasma jet device to investigate chemical species produced in ultrapure water in a polytetrafluoroethylene (PTFE) vessel.

We developed a handheld APPJ device for wound healing. As shown in Fig 1, the APPJ device consists of two ring-shaped copper electrodes (length of 5 mm), an alumina tube (inner diameter of 0.8 mm and outer diameter of 1.2 mm, SSA-S, Al_2_O_3_ >96%, Nikkato Corp., Japan), and a cylindrical acrylic vessel (outer diameter of 24 mm). Two ring-shaped electrodes are fixed to the alumina tube. The distance between these two electrodes is 4 mm. A low frequency AC high-voltage power source (100-C-9HEP2, LECIP corp., Japan) is connected to the upper ring-shaped electrode via a silicon rubber-insulated wire, while the lower ring-shaped electrode is connected to the ground. The two ring-shaped electrodes are covered by epoxy resin to prevent abnormal discharge outside of the ring electrodes. The length of the alumina tube nozzle from the lower edge of the lower ring electrode to the edge of the alumina tube outlet is 54 mm. In the present study, the frequency of the AC high-voltage source was approximately 17 kHz. A peak-to-peak voltage of 18 kV was adjusted with a varying AC input voltage using a voltage slider (YAMABISHI, Voltage slider, S-130-15, Japan). A low-frequency high voltage was applied after the introduction of argon gas from the upper end of the alumina tube at a flow rate of 0.3 standard liters per minute (slm). UP3 grade (99.999% Ar, O_2_ <0.2 ppm, CO_2_ <1.0 ppm, and H_2_ <1.0 ppm) argon was used as the working gas. The gas flow rate was controlled using a mass flow controller unit (SEC-E40, HORIBASTEC, Japan) and monitored by a digital monitor with a controller (PE-D20, HORIBASTEC, Japan). During the generation of APPJ, the high voltage applied was monitored in real time using an oscilloscope (WaveAce 1012, LeCroy Crop., USA) connected to a high-voltage probe (P6015A, TEKTRONIX, USA). The plasma plume of the APPJ device was not clearly observed by the naked eye due to the long nozzle length; therefore, the resulting plasma plume did not make direct contact with wounds. It is important to note that the gas flow rate and treatment time were low to prevent the wound area drying during treatment.

### Detection of RONS generated in liquid by APPJ irradiation

To investigate the generation of RONS in liquid, the concentrations of H_2_O_2_, nitrate (NO_2_^-^), and nitrite (NO_3_^-^) were measured in a vessel containing ultrapure water irradiated by APPJ. The vessel was made from polytetrafluoroethylene (PTFE). The inner diameter, height, and thickness of the PTFE vessel were 14, 9.8, and 1 mm, respectively. Ultrapure water was produced by a water purification system (Smart2Pure6 UV/UF, 50129887, Thermo Scientific Barnstead, USA). The volume of ultrapure water used in the PTFE vessel was 1.4 ml. The distance between the top edge of the PTFE vessel and the alumina nozzle edge was fixed at 5 mm.

The H_2_O_2_–colorimetric method, in which titanyl ions react and create yellow-colored pertitanic acid [13], was performed using a UV–Vis spectrophotometer (U-4100, Hitachi High Tech. Corp., Japan) to quantify H_2_O_2_ concentrations. Ti(SO_4_)_2_ solution (30wt%, CAS RN. 27960-69-6, Fujifilm Wako Chemicals, Japan) was diluted to 5wt% using ultrapure water for the H_2_O_2_–colorimetric method. One-milliliter liquid samples of APPJ-treated ultrapure water were collected and mixed with 1 mL of the titanium oxysulfate-sulfuric acid complex (TiOSO_4_) solution [14]. The absorbance of the mixed complex solution was measured at a wavelength of 410 nm, and converted to a H_2_O_2_ concentration using a calibration curve generated with a stock solution of H_2_O_2_ (30wt%, CAS RN. 7722-84-1, Fujifilm Wako Chemicals, Japan). In cases in which absorbance was higher than 1.0, liquid samples were diluted with ultrapure water prior to the addition of 1 mL of TiOSO_4_ solution and measured absorbance was multiplied by the dilution factor.

High performance liquid chromatography (HPLC, Prominence, Shimadzu Corp., Japan) was used to quantify NO_3_^-^ and NO_2_^-^ concentrations. An APPJ-treated ultrapure water sample of 1.0 ml was prepared and added to a polyethylene vial (Sample cup II A, Shinwa Chemical Industries Ltd., Japan). Vials were placed in an auto sampling system (STL-10Ai, Shimadzu Corp., Japan). Calibration curves for ion chromatography (IC) were prepared using various dilutions of an anion mixed standard solution (01849–96, Kanto Kagaku Corp., Japan). All liquid samples were analyzed on the same day without storage after the APPJ treatment.

Table 1 shows the analytical conditions of IC used in the present study. The conditions employed to measure RONS are provided in Table 2.

**Table 1:** Ion chromatography operating conditions.

Column	Anion	: Shim-pack IC-SA2(G)
Eluent	NaHCO_3_	: 12 mM
	Na_2_CO_3_	: 0.6 mM
Column oven	CTO-20AC sp	: 40°C
Flow rate of the eluent	1.0 mL/min	
Amount of the sample	500 μL	
Detector	CDD-10A sp	

**Table 2:** Experimental conditions and settings for reactive species measurements produced by the plasma device.

Room temperature	24.4°C	Room humidity	48%
Supply voltage	V_p-p_ = 18.0 kV_p-p_	Frequency	16.7 kHz
Distance between electrodes		4 mm	
Gas flow rate		Argon: 0.3 slm	
Conditions		Irradiation times: 2, 4, 5, 6, 8, 10 min	
		Irradiation distance: 5 mm	
Liquid		Ultrapure Water	
Liquid volume		1.4 mL	
Alumina tube		*Φ*_out_ 1.2 mm	*Φ*_in_ 0.8 mm

### Animals

Thirty 10-week-old male C57BL/6j mice with normal blood glucose levels and 30 10-week-old male db/db mice (C57BLKS/J Iar-+Lepr^db^/+Lepr^db^) as a diabetic model (Sankyo Lab Service Co., Tokyo, Japan) were used in experiments. Animals were individually caged in a temperature-controlled room (25.0 ± 2.0°C) with lights on between 08:45 and 20:45 and free access to water and chow. All animal experiments conducted in the present study were reviewed and approved by the Animal Experiment and Use Committee of Kanazawa University and performed in accordance with the Guidelines for the Care and Use of Laboratory Animals of Kanazawa University, Japan (AP-194100).

### Wounding and treatment

Mice were kept and fed until 10 weeks of age. Before the wounding day, mice were anesthetized by inhalational anesthesia using 1.5% isoflurane (Wako, Tokyo, Japan) in 1.5 L O_2_/min connected to the acrylic box [15–17] and the dorsum was shaved. Mice were divided into the following main groups: normal mice (DM- group) and diabetic mice (DM+ group), and then subdivided based on the treatment received. Normal mice treated with the plasma jet and a hydrocolloid dressing (DM-Plasma), normal mice treated with argon gas and a hydrocolloid dressing (DM-Argon), natural healing for normal mice treated only with a hydrocolloid dressing (DM-Hydrocolloid), diabetic mice treated with the plasma jet (DM+plasma), diabetic mice treated with argon gas (DM+Argon), and natural healing for diabetic mice treated only with the hydrocolloid dressing (DM+Hydrocolloid). We used the treatment with argon gas only to confirm that plasma was solely promoting wound healing.

On the wounding day, the dorsum was disinfected with 70% ethanol, and two circular full-thickness skin wounds (Ø4 mm), including the *panniculus carnosus* muscle, were made on both sides of the dorsum using a Kai sterile disposable biopsy punch (Kai Industries Co., Ltd., Gifu, Japan) under inhalational anesthesia. Each wound received the APPJ treatment for 60 seconds, and the irradiation distance from the alumina nozzle tip was 5 mm. The applied APPJ irradiation time, distance, and gas flow rate stably generated APPJ and supplied the APPJ effluent to the wound directly without drying the wound area. This treatment was performed once daily.

Wounds were covered by a hydrocolloid dressing (Tegaderm; 3M Health Care, Tokyo, Japan) to maintain a moist environment and wrapped with sticky bandages (Skinergate^™^; Nichiban, Tokyo, Japan), which were replaced every day according to previous studies by Mukai et al. [18, 19]. Body weights and hydrocolloid dressing weights were measured daily before and after dressing to assess the weight of exudate produced from the wound. The non-fasting blood glucose levels of normal and diabetic mice were monitored every two days using a glucose rapid test stick (Nipro CareFast R; Nipro, Osaka, Japan) and blood samples were obtained by a small prick of the mouse tail.

### Wound observations

The wounding day was designated as day 0. The progression of wound healing was monitored daily until day 14 (normal mice) and day 21 (diabetic mice) under inhalational anesthesia. Wound edges were traced daily on polypropylene sheets by making a drawing of the wound area pattern on the polypropylene sheet using a permanent marker, and images of wounds were also taken. Traces on the sheets were captured with a scanner onto a personal computer using Adobe Photoshop Elements 11.0 (Adobe System Inc., Tokyo, Japan), and wound areas were calculated using ImageJ (National Institutes of Health, Bethesda, Maryland, USA) image analysis software according to previous studies. The wound area was expressed as the daily ratio of the wound area to the initial wound area on day 0 when the wound was made [18, 19].

### Tissue collection

In the normal group, we sacrificed 5 mice for each treatment group on days 7 and 14. In the diabetic group, we sacrificed 3 mice for each treatment group on days 7 and 14, and 4 mice for each treatment group on day 21. Mice were euthanized by inhalational anesthesia on days 7, 14, and 21 after wounding. The wound and surrounding intact skin (epidermis, dermis, and subcutaneous tissue), the area of which was approximately 10% of the wound, were harvested, and each wound sample was bisected at the wound center. Wound tissue was spread on and stapled to polypropylene sheets to avoid over-contraction of the samples, and then fixed for approximately 15 h in 4% paraformaldehyde solution in 0.01 M phosphate buffer, pH 7.4. Sheets were rinsed in 0.01 M PBS for approximately 15 h. Samples were then dehydrated in an alcohol series, cleared in xylene, and embedded in paraffin (Sakura Finetek, Japan) to prepare 5-μm-thick serial sections. These procedures were performed according to previously reported methods [8].

### Histological and immunohistological staining

Five-micrometer-thick paraffin sections were stained with hematoxylin and eosin or subjected to immunohistology with an anti-α-smooth muscle actin (α-SMA) antibody (ab5694, Abcam Japan, Tokyo, Japan). To detect the primary antibody, sections for the anti-α-SMA antibody were incubated with the Dako Envision+ system HRP-labeled polymer anti-rabbit (ready to use) (Dako North America, California, USA). Negative control slides were obtained by omitting the primary antibody [18, 19]. The anti-α-SMA antibody was used in the present study because α-SMA is present in the pericytes covering blood capillaries and in smooth muscles in blood vessel walls [20, 21].

### Histological observations

Images were imported onto a computer using a digital microscopic camera (DP2-BSW Olympus, Japan). Measurements of the percentage of re-epithelialization on days 7, 14, and 21 were performed using DP2-BSW software. The number of positively stained blood vessel cells on days 7, 14, and 21 was assessed at five wound sites of granulation tissue, two sites near the two wound edges, and three sites around the center of granulation tissue using the 20× objective magnification, and the value was divided by the whole area of these five sites. The total area of the five sites was calculated on the DP2-BSW monitor [19].

### Statistical analysis

Data are shown as the mean ± standard deviation (SD) and the statistical analysis was performed by a paired t-test or one-way ANOVA and Tukey–Kramer multiple comparison test with SPSS 25.0 (SPSS Inc., Chicago, USA). p <0.05 was considered to be significant.

## Results

### Measurement of RONS concentrations

**Fig 2:**
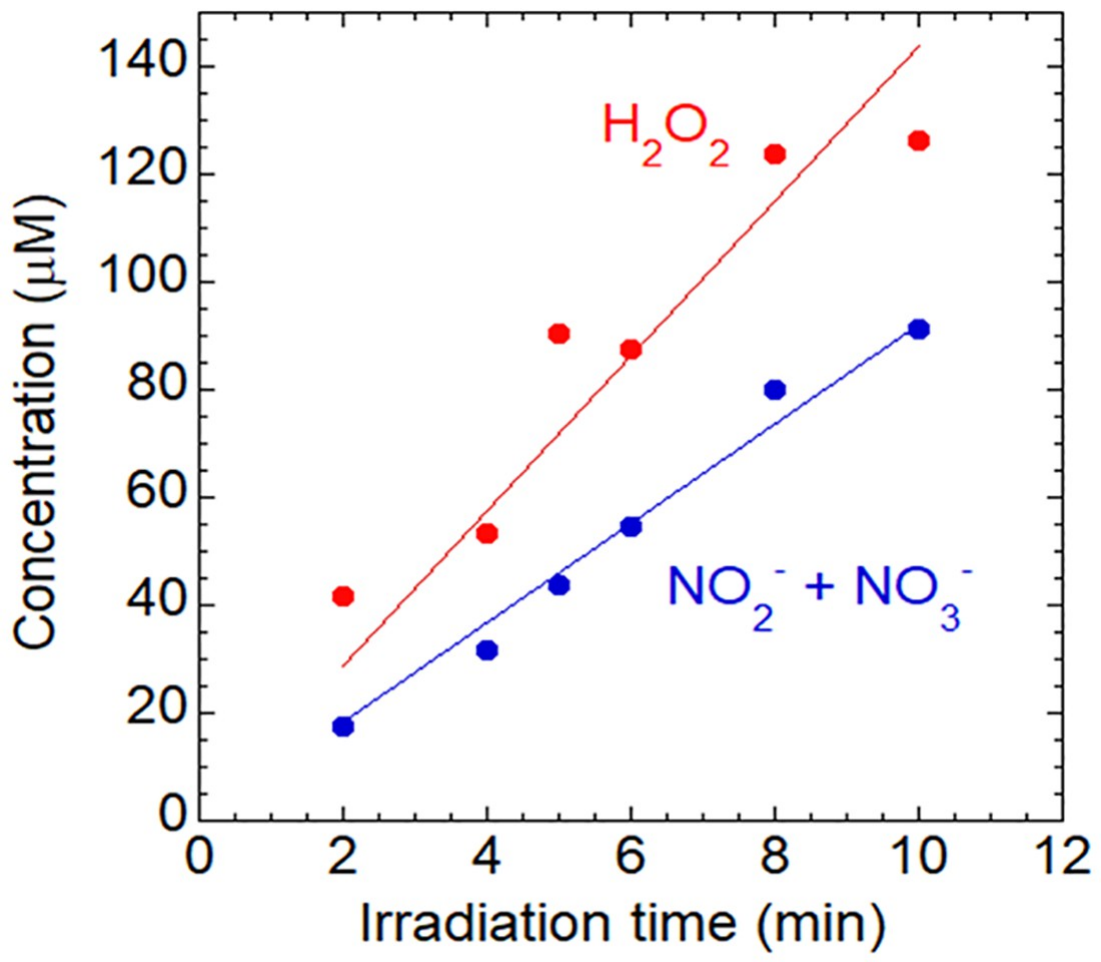
H_2_O_2_ and the sum of NO_2_^-^ and NO_3_^-^ concentrations in APPJ-irradiated ultrapure water as a function of the exposure time.

**Fig 3:**
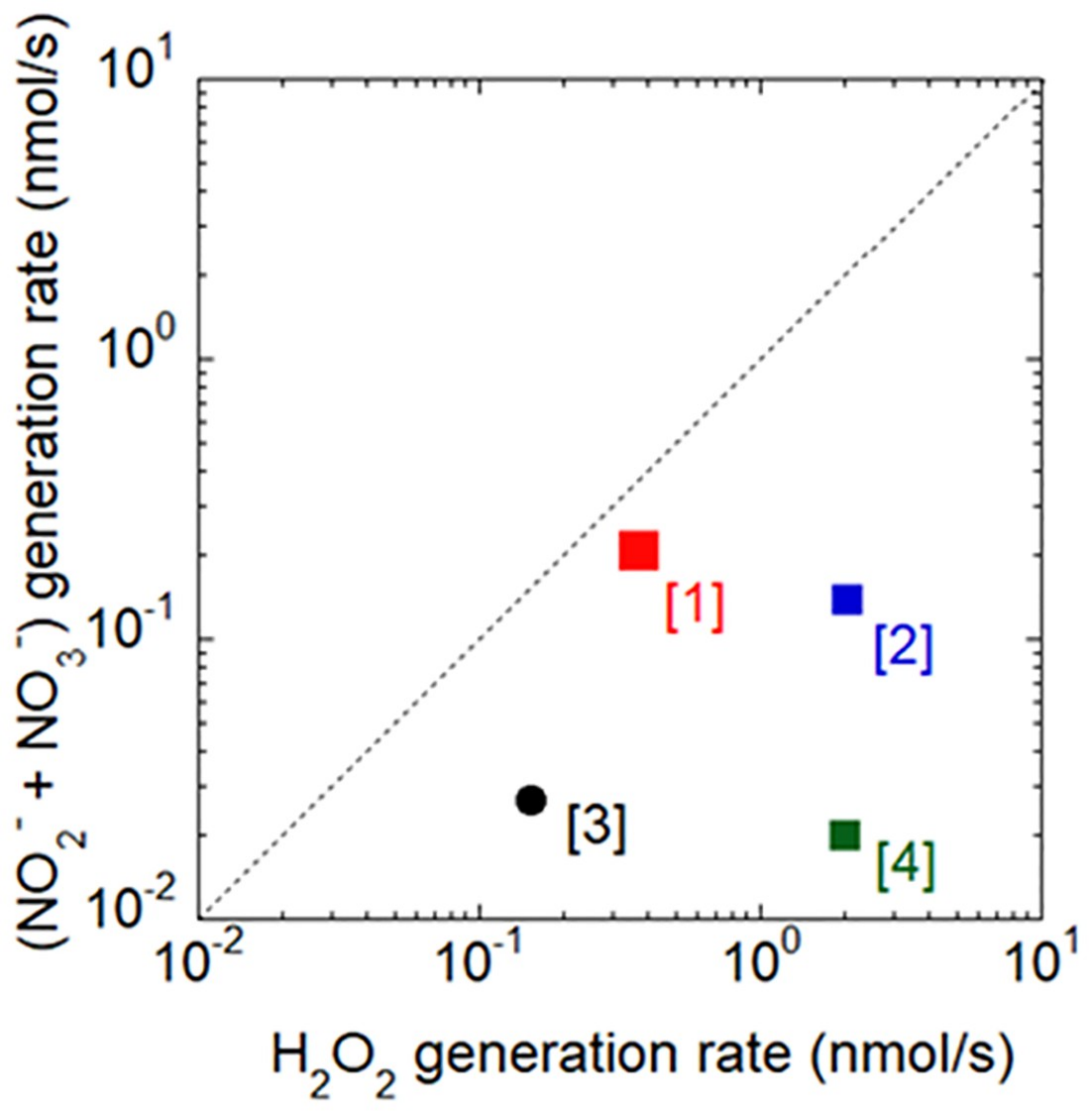
Correlations between generation rates of H_2_O_2_ and the sum of NO_2_^-^ and NO_3_^-^ produced in water in the present and previous studies. (1) The present study, (2) Cheng et al. [22], (3) Duchesne et al. [23], and (4) Nasruddin et al. [24].

Fig 2 shows the concentration of H_2_O_2_ and the sum of NO_2_^-^ and NO_3_^-^ concentrations as a function of the APPJ treatment time. Longer treatment times were associated with the concentration of H_2_O_2_ and the sum of NO_2_^-^ and NO_3_^-^ concentrations monotonically increased. The generation rates of H_2_O_2_, *G*_H2O2_, and the sum of NO_2_^-^ and NO_3_^-^ concentrations, *G*_NOx-_, were evaluated assuming a linear function with a least-squares approximation. A similar approach was previously applied to obtain *G*_H2O2_ and *G*_NOx-_. Fig 3 shows *G*_NOx-_ as a function of *G*_H2O2_ including previous findings [closed red squares are for the present study, and closed blue squares [22], closed black circles [23], and closed green squares [24] for previous studies]. The plume of our previously developed APPJ [24] made direct contact with the liquid surface, whereas that of the present APPJ did not. *G*_NOx-_ of the present APPJ device was similar to the value reported by Cheng et al. [22], whereas *G*_H2O2_ was 5-fold lower.

### Wound size observations, blood glucose levels, body weights, exudate weights, and wound areas

Blood glucose levels were stable each day. Blood glucose levels ranged between 100–200 mg/dL in normal mice (DM- group) and were higher than 500 mg/dL in db/db mice (DM+ group).

**Fig 4:**
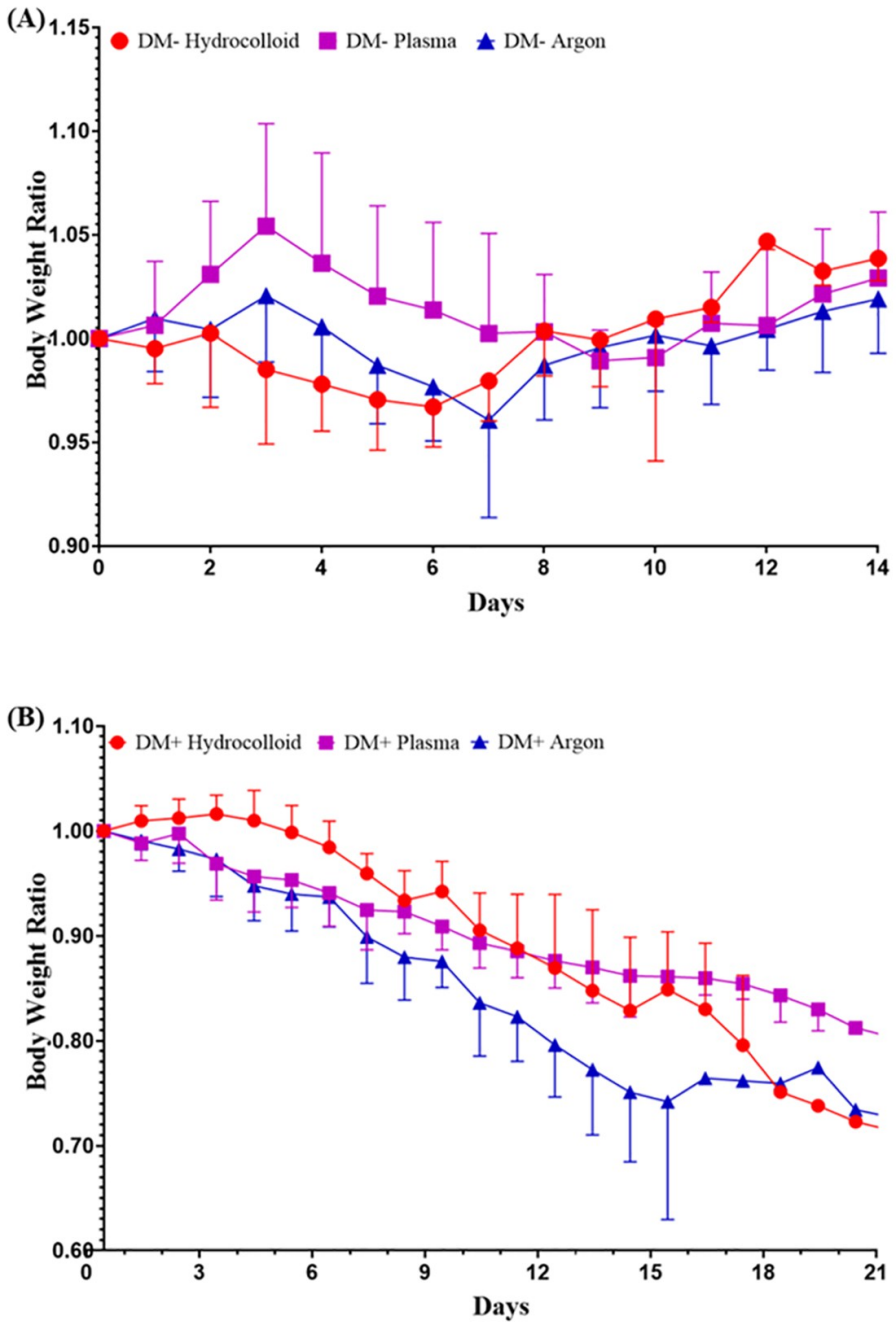
Daily body weight ratio. A comparison of daily body weights with initial body weights. (A) The body weight ratio in the DM- group is presented as the mean ± SD of five mice and (B) the body weight ratio in the DM+ group as the mean ± SD of four mice.

Body weight was measured throughout the study period and compared to that before wounding (Fig 4). Body weight continued to increase in the DM- group until days 3–4 after wounding, but then decreased. Body weight increased again on days 7–10 and surpassed the initial body weight on the last day of measurements. After wounding, body weight in the DM+ group decreased and was lower than the initial body weight on the last measurement day.

**Fig 5:**
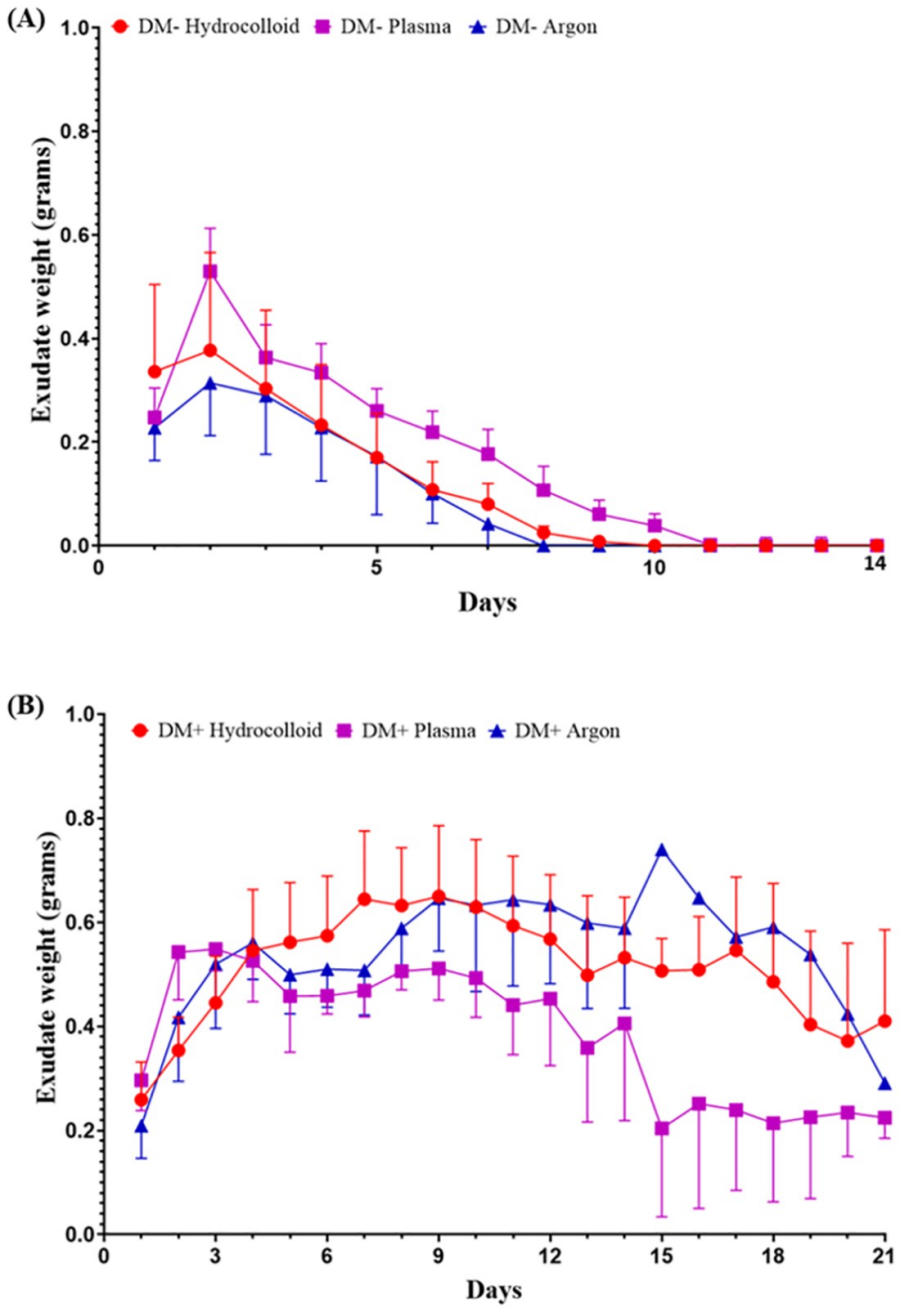
Exudate weights. Exudate weights in each group were recorded daily by measuring the weight (grams) of the hydrocolloid dressing. (A) Exudate weights in the DM- group are presented as the mean ± SD of ten wounds, and (B) exudate weights in the DM+ group as the mean ± SD of eight wounds.

Exudate weights varied and fluctuated in the DM+ group, but showed the same pattern in the DM- group (Fig 5). In the DM- group, exudate weights were the highest on day 2 and then decreased. In the DM+Plasma group, exudate weights were the highest on day 3. In the DM+Argon and DM+Hydrocolloid groups, exudate weights continued to increase and fluctuate during the three-week observation.

**Fig 6:**
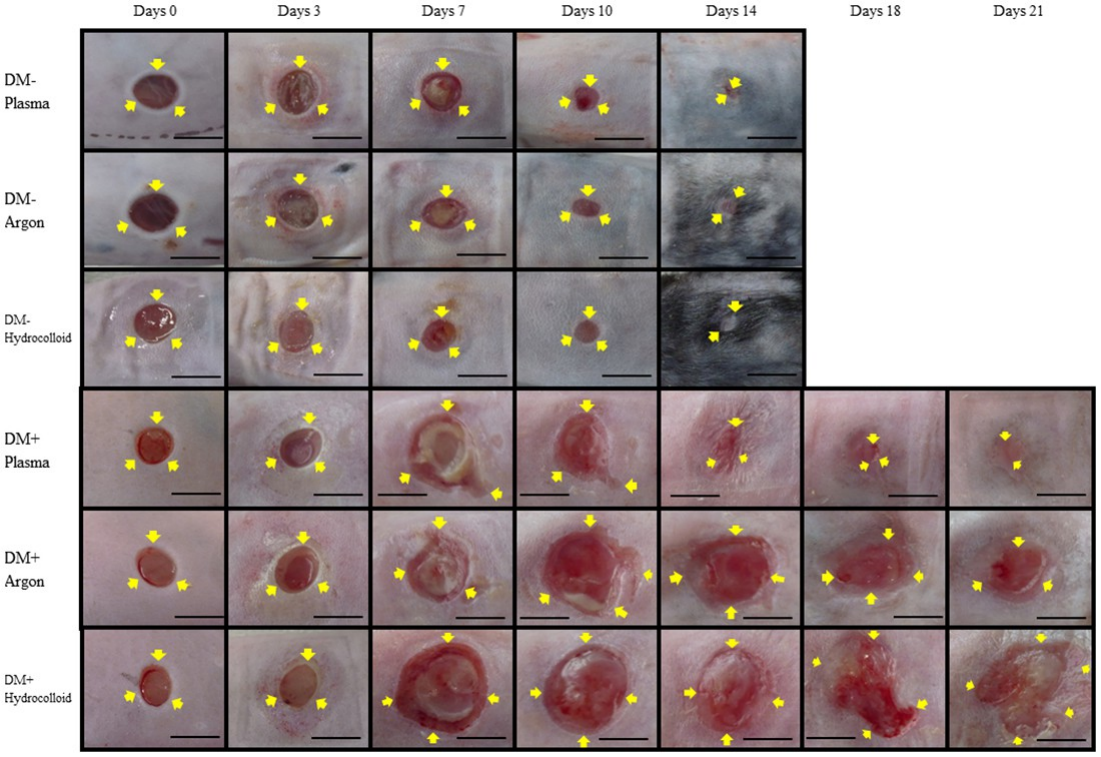
Wound healing observations in normal and diabetic mice. Wounds of 4 mm in diameter were made on the dorsum, and healing was recorded by photography. The wound edge is indicated by arrows. Bar, 5 mm.

Wound size evaluations were performed in every group from days 0 to 14 for the DM- group and from days 0 to 21 for the DM+ group, as shown in Fig 6. Wound areas generally increased during the early wound stage and then decreased until the end of the observation period. Wound sizes markedly increased until day 4.

Wound areas in the DM- group: In the DM-Plasma group, wound areas increased from days 1 to 3 (ratio of the wound area to the initial wound area on day 3: 1.07 ± 0.15), and then gradually decreased until wound closure on day 14 (0.18 ± 0.05). A similar pattern was observed in the DM-Argon and DM-Hydrocolloid groups, namely, an increase in the wound area from day 1 after wounding, with a peak on day 2 (1.54 ± 0.55 and 1.26 ± 0.22 respectively). Wound areas in the DM-Argon and DM-Hydrocolloid groups continuously decreased until wound closure, but were larger than those in the DM-Plasma group on day 14 (0.24 ± 0.12 and 0.30 ± 0.09 respectively). In contrast, wound areas in the DM+Hydrocolloid group markedly increased until day 4 (2.41 ± 0.82) and then decreased until day 21, but were still larger than the initial wound area (1.20 ± 0.03). A similar pattern was observed in the DM+Argon group; an increase in the wound area from days 1 to 3 of observations (1.91 ± 0.24), followed by a continual decrease until day 21 to a similar area as that of the initial wound (0.93 ± 0.10) without wound closure. In the DM+Plasma group, the wound area increased on day 1, followed by a decrease to a similar area as that of the initial wound on day 7 (1.02 ± 0.20). On day 14, the wound area was a similar size as that in the DM-Plasma group (0.23 ± 0.09). At the end of observations on day 21, the wound area was smaller than that in the DM-Plasma group (0.11 ± 0.07).

**Fig 7:**
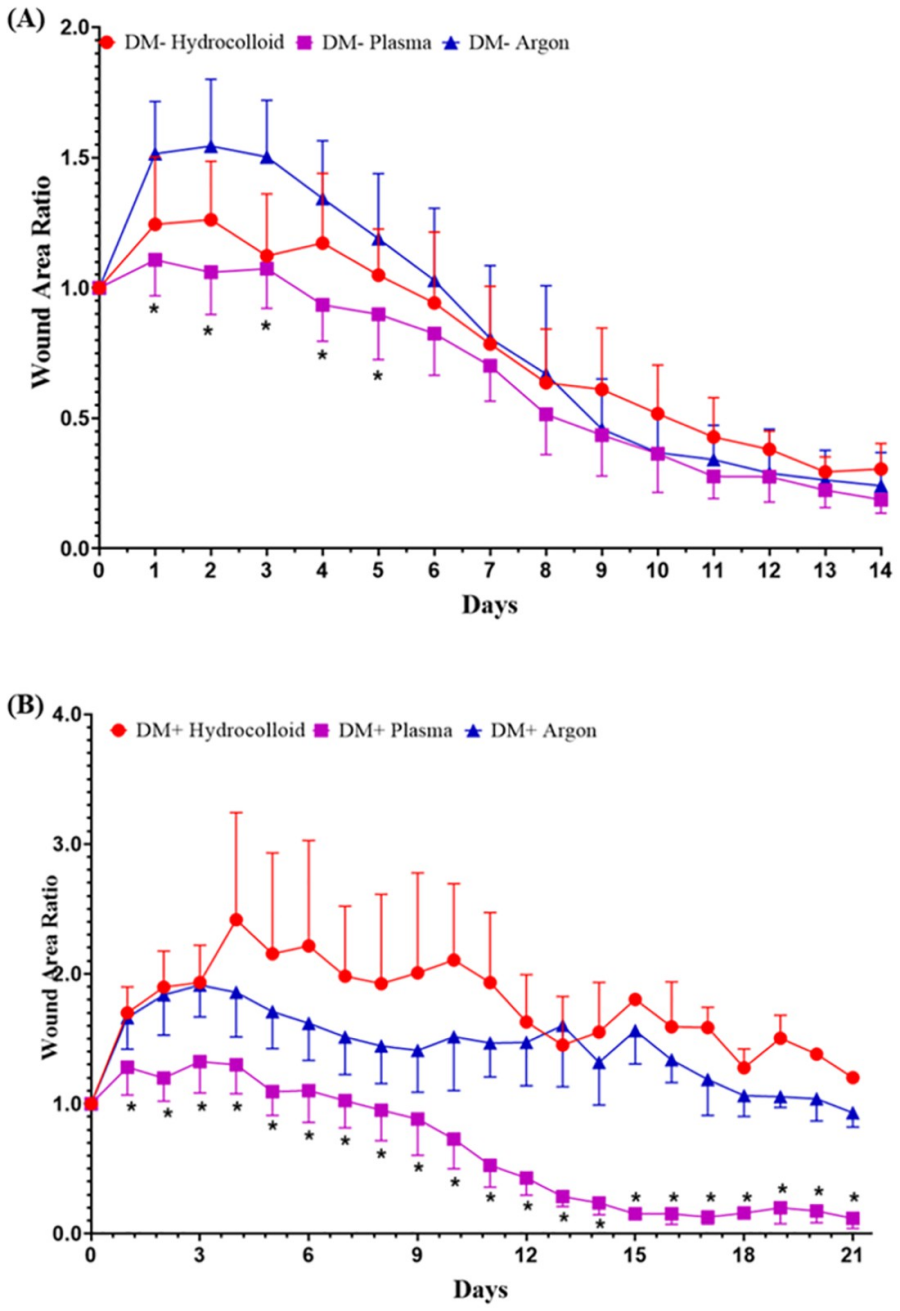
Comparison of wound area ratios in normal and diabetic mice. The ratios of wound areas to the initial area on day 0 are shown as line graphs for each day. (A) The wound area ratio in the DM- group is presented as the mean ± SD of ten wounds, and (B) the wound area ratio in the DM+ group as the mean ± SD of eight wounds. Values are expressed as the mean ± SD, ANOVA, Tukey-Kramer *p<0.01.

Fig 7 shows that the wound area ratio was significantly higher in the DM+Hydrocolloid and DM+Argon groups than in the DM+Plasma group on days 1–21 (p<0.01). Daily wound area ratios were smaller in the DM-Plasma group than in the DM-Argon group until day 14, with significant differences being observed on days 1–5 (p<0.01). No significant differences were observed in wound area ratios between the DM+Plasma and DM-Plasma groups, the difference occurred on days 3, 4, and 7 (p<0.05).

### Histological analyses

**Fig 8:**
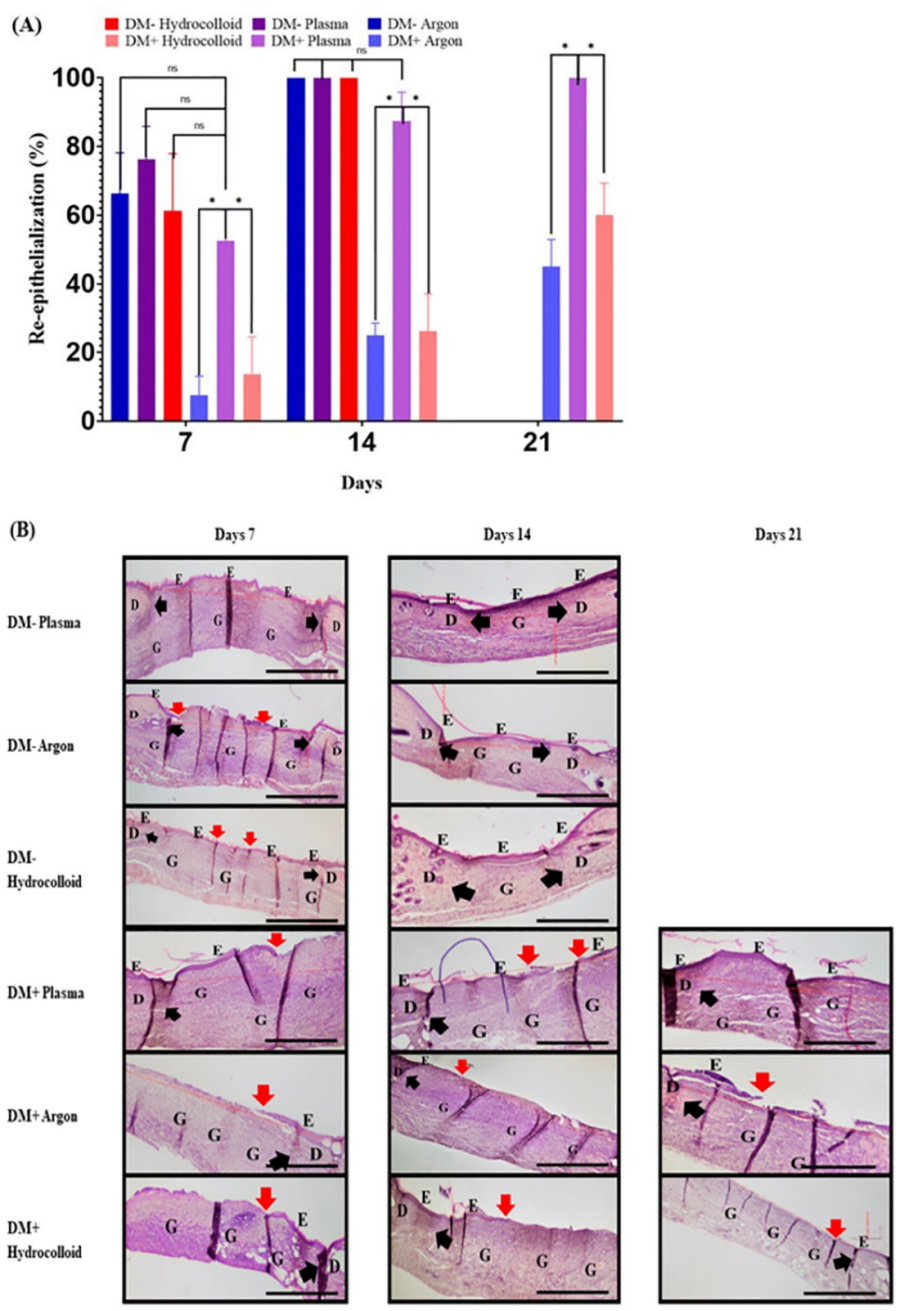
Percentage of re-epithelialization in wound healing. The percentage of re-epithelialization in wound healing on the skin of the dorsum. (A) The percentages of re-epithelialization in the DM- and DM+ groups on days 7, 14, and 21 are shown in box graphs. Values are expressed as the mean ± SD, ANOVA, Tukey-Kramer *p<0.01. (B) Histological features of wounds stained by hematoxylin and eosin. Red arrows indicate the tip of the re-epithelium (E) covering the wound surface. Block arrows indicate the edge of the wound, the boundary between the normal dermis (D) and granular tissue (G). The distances between the edges of wounds were markedly shorter in DM- mice than in DM+ mice. Wound surfaces on DM- mice on day 14 were already covered by a re-epithelium. Although wound surfaces in the DM+Argon and DM+Hydrocolloid groups were not yet covered by a re-epithelium on day 21, those of the DM+Plasma group were. Scale bar, 500 μm.

The percentage of re-epithelialization during wound healing was evaluated on days 7 and 14 for the DM- group and on days 7, 14, and 21 for the DM+ group (Fig 8). On day 21, all wound surfaces were covered by an epithelium. Furthermore, in comparisons of the percentage of re-epithelialization, no significant differences were observed between the DM+Plasma group and all DM-groups on days 7 and 14 (p>0.05). On day 21, the percentage of re-epithelialization was significantly higher in the DM+Plasma group than in the DM+Argon and DM+Hydrocolloid groups (p<0.01). The new epithelium generally extended from the wound edges and had completely covered the whole wound by the last measurement day, except for the DM+ Hydrocolloid and DM+Argon groups. Although the new epithelium slowly grew from the wound edges, it did not cover the entire wound from days 14 to 21 in the DM+Hydrocolloid and DM+Argon groups.

New blood vessels were observed in the wound area on days 7, 14, and 21 (Table 3). In the DM- and DM+Plasma groups, new blood vessels were more frequently observed in granulation tissue on day 7 and decreased on day 14. On the other hand, in the DM+Argon and DM+Hydrocolloid groups, the number of new blood vessels in granulation tissue was low on day 7, but continued to increase until day 21.

**Table 3:** Number of new blood vessels.

	DM-Plasma	DM-Argon	DM-Hydrocolloid	DM+Plasma	DM+Argon	DM+ Hydrocolloid
Day 7	15.4 ±2.1*^#^	11.6 ±4.3*^#^	14.2 ±3.5*^#^	12.8 ±3.2	4.6 ±1.1^a^	4.0 ±0.8^a^
Day 14	7.8 ±0.8^A^	10.8 ±3.2	12.4 ±1.5	11.2 ±1.4	9.6 ±2.9	8.6 ±2.3
Day 21				8.4 ±2.1	15.4 ±1.5^aA^	13.8 ±1.5^aA^

Values are expressed as the mean ± SD. The lowercase letter (a) indicates significantly different among DM+Argon or DM+Hydrocolloid groups and DM+Plasma group. The uppercase letter (A) indicates significantly different within groups compared with on day 7. The (*) symbols indicate significantly different between DM- groups and DM+Argon group, and the (#) symbols indicate significantly different between DM- groups and DM+Hydrocolloid group. ANOVA, Tukey-Kramer and paired t-test p<0.05.

A significant difference was observed in the number of new blood vessels between the DM+Plasma and DM+Argon and DM+Hydrocolloid treatment on days 7 and 21. A significant difference was also observed in decreased new blood vessels count on days 7 compared to days 14 in the DM-Plasma group. In the DM+Plasma group, we found that the new blood vessels tended to decrease from days 7 until 21, but did not differ statistically. An opposite result was observed on DM+Argon and DM+Hydrocolloid group, which significantly increased when comparing days 7 and days 21.

**Fig 9:**
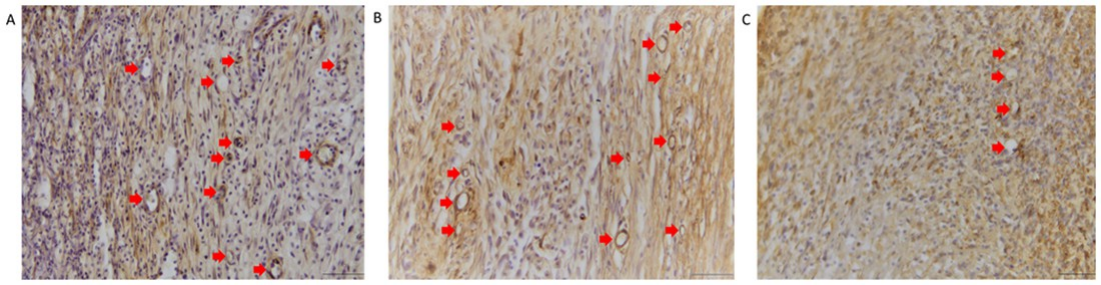
Microscopic view of new blood vessel formation in areas of granulation. Myofibroblasts and pericytes were stained brown because they contain α-SMA. Blood vessels are circular in shape and their walls containing pericytes are brown (arrows). Therefore, it is possible to distinguish between myofibroblasts and blood vessels (A) The formation of new blood vessels in the DM-Plasma group on day 7. (B) The formation of new blood vessels in the DM+Plasma group on day 7. (C) The formation of new blood vessels in the DM+Hydrocolloid group on day 7. Scale bar, 50 μm. Anti-α-SMA stain.

The number of new blood vessels did not differ significantly between the DM+Plasma and DM- groups in all treatments on days 7 or 14. The figure of the formation of new blood vessels on days 7 from the anti-α-SMA stain result is presented in Fig 9.

## Discussion

The present results demonstrated that H_2_O_2_ concentrations and the sum of NO_2_^-^ and NO_3_^-^ concentrations increased in APPJ-irradiated ultrapure water. Longer treatment times were associated with H_2_O_2_ and the sum of NO_2_^-^ and NO_3_^-^ concentrations monotonically increased. These changes indicated that RONS production induced by the APPJ device varied depending on the device settings. These results are consistent with previous findings reported by Kinandana et al. [25] showing increases in H_2_O_2_ concentrations with a longer jet plasma exposure time. Kim and Chung [26] also reported changes in RONS concentrations with different APPJ settings.

The APPJ device used in the present study produced higher concentrations of NO_2_^-^ and NO_3_^-^ and a lower concentration H_2_O_2_ than those in other studies [22–24]. This difference in the rate of RONS production contributed to the unique effects observed on the wound healing process. Pre-clinical findings suggested that some gas plasma-derived ROS/RNS mixtures are more beneficial than others for specific biological outcomes. According to Bekeschus [27], three different feed gas supplements of a plasma jet exerted individual effects. Hypothetically, plasma that is rich in NO contains a small amount of a mixture of the hydroxyl (OH) radical and atomic oxygen, and, thus, promotes the closure of wounds and angiogenesis and also functions as an antimicrobial agent, albeit not a potent one.

The present study utilizes RONS produced by APPJ for wound healing and was done using normal mice and diabetic mice both with and without plasma treatment. Since wound healing is delayed under diabetic conditions [3, 27, 28], we herein used a non-thermal plasma jet that produces RONS to treat wounds. RONS from plasma play essential roles in the interactions between plasma and targeted tissues. Based on previous findings, APPJ, which produces RONS, such as NO and H_2_O_2_, may effectively promote wound healing [11, 12, 29, 30]. Furthermore, current research uses non-thermal plasma under non-contact conditions; therefore, wounds are not directly exposed to the plasma plume, which prevents the adverse events associated with charged particles. Although non-thermal plasma that produces reactive species in liquid at an appropriate intensity may promote wound healing, it may also exert detrimental effects on cells and tissues if treatment is prolonged or higher plasma densities are applied [31–34]. A low density plasma treatment may activate the signaling pathways that enhance cell proliferation and tissue regeneration, while a high density plasma treatment may produce higher concentrations of RONS. The resulting oxidative stress disrupts redox signaling, damages biomolecules, such as membrane lipids, proteins, and DNA [11], and induces apoptosis in cancer cells by increasing intracellular ROS and activating the corresponding ROS-based death pathways [12].

The effects of the non-contact treatment in the present study were monitored by the progression of wound healing. Wound size observations revealed the prominent advantages of using plasma for wound healing, particularly under diabetic conditions. Wound healing with the plasma treatment from the early wound phase was superior to that with the argon treatment or natural healing. Lower exudate weights in the DM+Plasma group than in the DM+Argon and DM+Hydrocolloid groups in the middle and last days of monitoring were an excellent indicator of wound healing in the inflammation phase. The inflammation phase was shorter in the DM+Plasma group, indicating the more rapid progression of this phase to the proliferative phase. A previous study reported the highest rate of exudate production during the inflammatory phase, followed by decreases with the progression of healing [35]. Exudate weights in the DM+Plasma group peaked almost simultaneously with those in all DM- groups on day 2. This similar peak indicated that the time course of the inflammation phase did not significantly differ between these groups. Exudate production during the inflammation phase is attributed to the permeability of capillaries. Tight gaps between cells and capillary walls and the porous carbohydrate-rich lining of capillaries play important roles in regulating the release of fluid, proteins, and cells into the surrounding tissues. Inflammatory mediators disrupt endothelial cells, which stimulates the release of fluid, proteins, and cells [36]. Although the mechanisms underlying decreases in the number of inflammatory cells, which accelerate wound healing, were not investigated in the present study, they were examined in detail in our previous study [37]. Based on the results obtained, we hypothesize that the current non-thermal plasma device under non-contact conditions reduced inflammation. The lower production of exudate in the present study is supported by previous findings reported by Rezaeinezhad et al. [38] showing that RONS generated by plasma attenuated inflammation under diabetic conditions. Both groups reported that the levels of inflammatory cytokines, such as IL-1α, IL-1β, IL-6, and TNF-α, were significantly lower in treated than in non-treated diabetic animals, and were close to normal ranges. Furthermore, we previously suggested that the inflammatory phase is a critical period in cutaneous wound healing, which is essential for clearing bacterial contamination and creating an environment that is conducive to subsequent events involved in tissue repair and regeneration [19]. Plasma treatment has also been shown to facilitate the eradication of bacteria, which was also confirmed in our previous study [39]. In another study by Amini [40], a randomized clinical trial in Iran on 44 patients with diabetic foot ulcers, helium plasma jet treatment decreased the production of IL-1, IL-8, IFN-γ, and TNF-α and reduced the antimicrobial burden.

The wound area did not increase during the inflammatory phase in wounds treated with non-thermal plasma under non-contact conditions. Better results for the wound area and ratio reduction were observed in the DM+Plasma group than in the DM+Argon and DM+Hydrocolloid groups. The wound area in the DM+Plasma group increased on day 1, and then decreased to a similar area as that of the initial wound on day 7. However, wound areas in the other groups remained large until the end of observations. This size difference indicates that wound closure was accelerated by the plasma treatment, and this may have been due to the generation of RONS by the current non-thermal APPJ device. This is consistent with the findings of a clinical trial by Mirpour et al. [41] in which a patient who received standard care with plasma treatment showed the accelerated wound healing of diabetic foot ulcers and a reduced bacterial load. This is also supported by a recent study [27], which demonstrated that plasma stimulated wound healing because ROS and RNS were released locally and interacted in the exposed site. This study further suggested that ROS and RNS are the most important components attributing to the redox biology of several biological effects that are induced by gas plasma. *In vitro* studies also demonstrated the direct impact of plasma on cell proliferation and migration [12]. RONS generated by the plasma device under atmospheric pressure have been suggested to increase blood oxygenation levels in tissue, which regenerates epidermal cells with mature differentiation, and granulation tissue with dense collagen deposition produced by fibroblasts [42].

The microscopic analysis showed that the percentage of re-epithelialization was higher in the DM+Plasma group and this was attributed to the shorter inflammatory response time, with the highest exudate weights being observed on day 3, which indicated that the proliferation stage occurred earlier in this group. The percentage of re-epithelialization significantly differed between the DM+Argon and DM+Hydrocolloid groups and interestingly resulted in a non-significant difference compared to those in the DM- group. Moreover, the results on re-epithelialization were consistent with those obtained from immunohistological staining with the anti-α-SMA antibody to detect new blood vessel formation in plasma-treated wounds, showing a significant difference in the number of new blood vessels that formed in diabetic mice. New blood vessels formed in granulation tissue on day 7, formed bridge-like structures in the wound area, and then decreased in number until day 14. In the present study, fully re-epithelialized wounds and the smaller number of new blood vessels on day 14 indicated that plasma promoted wound healing in the proliferation phase. These results suggest that plasma accelerated wound healing by stimulating tissue regeneration, including re-epithelialization and new blood vessel formation, via RONS delivered by the current APPJ device to the wound area. These results are consistent with previous findings by Chatraie et al. [43], showing that a non-thermal plasma treatment accelerated re-epithelialization in a pressure ulcer, and those by Lee et al. [44] of plasma promoting re-epithelialization through the arrangement of collagen and the regulation of inflammatory gene expression for the healing of burn wounds. The effects of RONS from plasma on wounds may be explained as follows: NO stimulates keratinocytes to secrete growth factors, cytokines, and proteases during the wound healing process, which facilitates ECM regeneration and promotes wound healing [45]. Besides NO, the current non-thermal APPJ device also produces OH radicals and H_2_O_2_, which plays a role in wound healing as an active secondary messenger for PDGF and VEGF [11]. In the cell proliferation phase, a low concentration of H_2_O_2_ was shown to promote the mobility of keratinocytes and enhance epidermal growth factor receptor activation and ERK1/2 phosphorylation, explaining its higher migration potential [46]. In the later remodeling phase, H_2_O_2_ up-regulated the expression of transforming growth factor-1 and enhanced the proliferation of fibroblasts [47]. In addition to its role in the re-epithelialization of wounds, plasma has been suggested to promote angiogenesis and new blood vessel formation and induce arterial vasodilation. These processes increase blood flow, which provides more nutrients in the circulation and promotes the transmigration of leukocytes [27]. This is consistent with previous findings showing that a plasma treatment increased vascularization and tissue oxygenation [48]. Since no significant differences were observed in the percentage of re-epithelialization or new blood vessel formation among plasma-treated wounds, argon-treated wounds, and natural healing on day 7 in the DM- group in the present study, the benefits of using non-contact plasma treatment remain unclear. Further studies are warranted.

In conclusion, the developed non-thermal APPJ device with non-contact treatment generated an appropriate amount of RONS that successfully accelerated wound healing by reducing the amount of exudate produced, accelerating wound closure through the promotion of re-epithelialization, and stimulating the formation of new blood vessels. The current non-thermal plasma treatment under non-contact conditions by the developed APPJ device may influence wound healing mechanisms at the microenvironmental level through the RONS produced.

## References

[1] Singer A, Clark R. Cutaneous Wound Healing. N Engl J Med. 1999;341(10):738–46. doi: 10.1056/NEJM199909023411006 10471461

[2] Tan WS, Arulselvan P, Ng SF, Mat Taib CN, Sarian MN, Fakurazi S. Improvement of diabetic wound healing by topical application of Vicenin-2 hydrocolloid film on Sprague Dawley rats 11 Medical and Health Sciences 1103 Clinical Sciences. BMC Complement Altern Med. 2019;19(1):1–16. 10.1186/s12906-018-2427-y . 30654793 PMC6337851

[3] Lan CCE, Wu CS, Huang SM, Wu IH, Chen GS. High-Glucose environment enhanced oxidative stress and increased interleukin-8 secretion from keratinocytes. Diabetes. 2013;62(7):2530–8. 10.2337/db12-1714 . 23423570 PMC3712048

[4] Martí-Carvajal AJ, Gluud C, Nicola S, Simancas-Racines D, Reveiz L, Oliva P, et al. Growth factors for treating diabetic foot ulcers. Cochrane Database Syst Rev. 2015;2015(10). 10.1002/14651858.CD008548.pub2 . 26509249 PMC8665376

[5] Xu K, Yu FSX. Impaired epithelial wound healing and EGFR signaling pathways in the corneas of diabetic rats. Investig Ophthalmol Vis Sci. 2011;52(6):3301–8. 10.1167/iovs.10-5670 . 21330660 PMC3109029

[6] Graves DB. Low temperature plasma biomedicine: A tutorial review. Phys Plasmas. 2014;21(8). 10.1063/1.4892534

[7] von Woedtke T, Reuter S, Masur K, Weltmann KD. Plasmas for medicine. Phys Rep. 2013;530(4):291–320. 10.1016/j.physrep.2013.05.005

[8] Wahyuningtyas ES, Iswara A, Sari Y, Kamal S, Santosa B, Ishijima T, et al. Comparative study on Manuka and Indonesian honeys to support the application of plasma jet during proliferative phase on wound healing. Clin Plasma Med. 2018;12(September):1–9. 10.1016/j.cpme.2018.08.001

[9] Bielefeld KA, Amini-Nik S, Alman BA. Cutaneous wound healing: Recruiting developmental pathways for regeneration. Cell Mol Life Sci. 2013;70(12):2059–81. 10.1007/s00018-012-1152-9 . 23052205 PMC3663196

[10] Sen CK, Roy S. Redox signals in wound healing. Biochim Biophys Acta—Gen Subj. 2008;1780(11):1348–61. 10.1016/j.bbagen.2008.01.006 18249195 PMC2574682

[11] Boeckmann L, Schäfer M, Bernhardt T, Semmler ML, Jung O, Ojak G, et al. Cold atmospheric pressure plasma in wound healing and cancer treatment. Appl Sci. 2020;10(19):1–16. 10.3390/app10196898

[12] Von Woedtke T, Schmidt A, Bekeschus S, Wende K, Weltmann KD. Plasma medicine: A field of applied redox biology. In Vivo (Brooklyn). 2019;33(4):1011–26. 10.21873/invivo.11570 . 31280189 PMC6689367

[13] Eisenberg GM. Colorimetric Determination of Hydrogen Peroxide. Ind Eng Chem—Anal Ed. 1943;15(5):327–8. 10.1021/i560117a011

[14] Wandell RJ, Wang H, Tachibana K, Makled B, Locke BR. Nanosecond pulsed plasma discharge over a flowing water film: Characterization of hydrodynamics, electrical, and plasma properties and their effect on hydrogen peroxide generation. Plasma Process Polym. 2018;15(6):1–16. 10.1002/ppap.201800008

[15] Hohlbaum K, Bert B, Dietze S, Palme R, Fink H, Thöne-Reineke C. Severity classification of repeated isoflurane anesthesia in C57BL/6JRj mice—Assessing the degree of distress. PLoS One. 2017;12(6):1–21. 10.1371/journal.pone.0179588 . 28617851 PMC5472303

[16] Navarro KL, Huss M, Smith JC, Sharp P, Marx JO, Pacharinsak C. Mouse Anesthesia: The Art and Science. ILAR J. 2021;00(00):1–36. 10.1093/ilar/ilab016 34180990 PMC9236661

[17] Szczȩsny G, Veihelmann A, Massberg S, Nolte D, Messmer K. Long-term anaesthesia using inhalatory isoflurane in different strains of mice—The haemodynamic effects. Lab Anim. 2004;38(1):64–9. 10.1258/00236770460734416 . 14979990

[18] Mukai K, Nakajima Y, Urai T, Komatsu E, Nasruddin, Sugama J, et al. 17β-Estradiol administration promotes delayed cutaneous wound healing in 40-week ovariectomised female mice. Int Wound J. 2014;13:636–44. 10.1111/iwj.12336 25132513 PMC7949953

[19] Mukai K, Urai T, Asano K, Nakajima Y, Nakatani T. Evaluation of effects of topical estradiol benzoate application on cutaneous wound healing in ovariectomized female mice. PLoS One. 2016;11(9):1–15. 10.1371/journal.pone.0163560 27658263 PMC5033238

[20] Busuioc CJ, Popescu FC, Mogoşanu GD, Lascǎr I, Pirici I, Pop OT, et al. Angiogenesis assessment in experimental third degree skin burns: A histological and immunohistochemical study. Rom J Morphol Embryol. 2011;52(3):887–95. . 21892535

[21] Qiu X, Wang J, Wang G, Wen H. Vascularization of Lando^®^ dermal scaffold in an acute full-thickness skin-defect porcine model. J Plast Surg Hand Surg. 2018;52(4):204–9. 10.1080/2000656X.2017.1421547 . 29320909

[22] Cheng KY, Lin ZH, Cheng YP, Chiu HY, Yeh NL, Wu TK, et al. Wound Healing in Streptozotocin-Induced Diabetic Rats Using Atmospheric-Pressure Argon Plasma Jet. Sci Rep. 2018;8(1):1–15. 10.1038/s41598-018-30597-1 . 30111887 PMC6093903

[23] Duchesne C, Frescaline N, Lataillade JJ, Rousseau A. Comparative study between direct and indirect treatment with cold atmospheric plasma on in vitro and in vivo models of wound healing. Plasma Med. 2018;8(4):379–401. 10.1615/PlasmaMed.2019028659

[24] Nasruddin, Nakajima Y, Mukai K, Komatsu E, Rahayu HSE, Nur M, et al. A Simple Technique to Improve Contractile Effect of Cold Plasma Jet on Acute Mouse Wound by Dropping Water. Plasma Process Polym. 2015;12(10):1128–38. 10.1002/ppap.201400236

[25] Kinandana AW, Sumariyah S, Nur M. Analysis of Plasma-activated Medium (PAM) in aqueous solution by an Atmospheric Pressure Plasma Jet (APPJ). MATEC Web Conf. 2018;197(January). 10.1051/matecconf/201819702013

[26] Kim SJ, Chung TH. Cold atmospheric plasma jet-generated RONS and their selective effects on normal and carcinoma cells. Sci Rep. 2016;6(December 2015):1–14. 10.1038/srep20332 . 26838306 PMC4738260

[27] Bekeschus S, von Woedtke T, Emmert S, Schmidt A. Medical gas plasma-stimulated wound healing: Evidence and mechanisms: Mechanisms of gas plasma-assisted wound healing. Redox Biol. 2021;46(July):102116. 10.1016/j.redox.2021.102116 34474394 PMC8408623

[28] Oluwafemi Omoniyi Oguntibeju. Type 2 diabetes mellitus, oxidative stress and inflammation: examining the links. Int J Physiol Pathophysiol Pharmacol. 2019;11(3):45–63. . 31333808 PMC6628012

[29] Kolumam G, Wu X, Lee WP, Hackney JA, Zavala-Solorio J, Gandham V, et al. IL-22R ligands IL-20, IL-22, and IL-24 promote wound healing in diabetic db/db mice. PLoS One. 2017;12(1):1–20. 10.1371/journal.pone.0170639 . 28125663 PMC5268431

[30] Lima MHM, Caricilli AM, de Abreu LL, Araújo EP, Pelegrinelli FF, Thirone ACP, et al. Topical insulin accelerates wound healing in diabetes by enhancing the AKT and ERK pathways: A double-blind placebo-controlled clinical trial. PLoS One. 2012;7(5):1–13. 10.1371/journal.pone.0036974 . 22662132 PMC3360697

[31] Weiss M, Barz J, Ackermann M, Utz R, Ghoul A, Weltmann KD, et al. Dose-Dependent Tissue-Level Characterization of a Medical Atmospheric Pressure Argon Plasma Jet. ACS Appl Mater Interfaces. 2019;11(22):19841–53. 10.1021/acsami.9b04803 . 31071258

[32] Braný D, Dvorská D, Halašová E, Škovierová H. Cold atmospheric plasma: A powerful tool for modern medicine. Int J Mol Sci. 2020;21(8). 10.3390/ijms21082932 . 32331263 PMC7215620

[33] Privat-Maldonado A, Bengtson C, Razzokov J, Smits E, Bogaerts A. Modifying the tumour microenvironment: Challenges and future perspectives for anticancer plasma treatments. Cancers (Basel). 2019;11(12):1–34. 10.3390/cancers11121920 31810265 PMC6966454

[34] Ahn HJ, Kim K Il, Kim G, Moon E, Yang SS, Lee JS. Atmospheric-pressure plasma jet induces apoptosis involving mitochondria via generation of free radicals. PLoS One. 2011;6(11):6–12. 10.1371/journal.pone.0028154 . 22140530 PMC3226649

[35] Schultz GS, Davidson JM, Kirsner RS, Bornstein P, Herman IM. Dynamic reciprocity in the wound microenvironment. Wound Repair Regen. 2011;19(2):134–48. 10.1111/j.1524-475X.2011.00673.x . 21362080 PMC3051353

[36] World Union of Wound Healing Societies (WUWHS). Consensus Document. Wound exudate: effective assessment and management. Wounds International. 2019. https://www.woundsinternational.com/download/resource/7732

[37] Nasruddin, Nakajima Y, Mukai K, Rahayu HSE, Nur M, Ishijima T, et al. Cold plasma on full-thickness cutaneous wound accelerates healing through promoting inflammation, re-epithelialization and wound contraction. Clin Plasma Med. 2014;2(1):28–35. 10.1016/j.cpme.2014.01.001

[38] Rezaeinezhad A, Mahdavi-Gharavia M, Talebi-Khoshmehra M, Mirmiranpour H, Ghomi H. Cold atmospheric plasma treatment: a novel method for diabetes mellitus therapy; a basic study. Plasma Med. 2021;11(3):19–30. 10.1615/plasmamed.2021040177

[39] Darmawati S, Rohmani A, Nurani LH, Prastiyanto ME, Dewi SS, Salsabila N, et al. When plasma jet is effective for chronic wound bacteria inactivation, is it also effective for wound healing? Clin Plasma Med. 2019;14(18):100085. 10.1016/j.cpme.2019.100085

[40] Amini MR, Sheikh Hosseini M, Fatollah S, Mirpour S, Ghoranneviss M, Larijani B, et al. Beneficial effects of cold atmospheric plasma on inflammatory phase of diabetic foot ulcers; a randomized clinical trial. J Diabetes Metab Disord. 2020;19(2):895–905. 10.1007/s40200-020-00577-2 33520811 PMC7843664

[41] Mirpour S, Fathollah S, Mansouri P, Larijani B, Ghoranneviss M, Mohajeri Tehrani M, et al. Cold atmospheric plasma as an effective method to treat diabetic foot ulcers: A randomized clinical trial. Sci Rep. 2020;10(1):1–9. 10.1038/s41598-020-67232-x . 32591594 PMC7319950

[42] Xu GM, Shi XM, Cai JF, Chen S Le, Li P, Yao CW, et al. Dual effects of atmospheric pressure plasma jet on skin wound healing of mice. Wound Repair Regen. 2015;23(6):878–84. 10.1111/wrr.12364 26342154

[43] Chatraie M, Torkaman G, Khani M, Salehi H, Shokri B. In vivo study of non-invasive effects of non-thermal plasma in pressure ulcer treatment. Sci Rep. 2018;8(1):1–11. 10.1038/s41598-018-24049-z . 29618775 PMC5884810

[44] Lee OJ, Ju HW, Khang G, Sun PP, Rivera J, Cho JH, et al. An experimental burn wound-healing study of non-thermal atmospheric pressure microplasma jet arrays. J Tissue Eng Regen Med. 2016;10(4):348–57. 10.1002/term.2074 26227832

[45] Pan S, Zhang S, Chen H. Low temperature plasma promotes the healing of chronic wounds in diabetic mice. J Phys D Appl Phys. 2020;53(18):1–10. 10.1088/1361-6463/ab7514

[46] Loo AEK, Wong YT, Ho R, Wasser M, Du T, Ng WT, et al. Effects of Hydrogen Peroxide on Wound Healing in Mice in Relation to Oxidative Damage. PLoS One. 2012;7(11). 10.1371/journal.pone.0049215 . 23152875 PMC3496701

[47] Wilgus TA, Bergdall VK, Dipietro LA, Oberyszyn TM. Hydrogen peroxide disrupts scarless fetal wound repair. Wound Repair Regen. 2005;13(5):513–9. 10.1111/j.1067-1927.2005.00072.x . 16176460

[48] Schmidt A, von Woedtke T, Vollmar B, Hasse S, Bekeschus S. Nrf2 signaling and inflammation are key events in physical plasma-spurred wound healing. Theranostics. 2019;9(4):1066–84. 10.7150/thno.29754 . 30867816 PMC6401410

